# Treat to transmural healing: how to incorporate intestinal ultrasound into the treatment of inflammatory bowel disease

**DOI:** 10.1259/bjr.20211174

**Published:** 2022-07-13

**Authors:** Aysha H. Al-Ani, Rose Vaughan, Britt Christensen, Robert V. Bryant, Kerri L. Novak

**Affiliations:** Department of Medicine, University of Melbourne, Melbourne, Australia; Department of Gastroenterology, The Royal Melbourne Hospital, Melbourne, Australia; Department of Medicine, University of Melbourne, Melbourne, Australia; Department of Gastroenterology, The Royal Melbourne Hospital, Melbourne, Australia; Department of Medicine, University of Melbourne, Melbourne, Australia; Department of Gastroenterology, The Royal Melbourne Hospital, Melbourne, Australia; Department of Gastroenterology, The Queen Elizabeth Hospital, Woodville, Australia; School of Medicine, University of Adelaide, Adelaide, Australia; Department of Gastroenterology, The University of Calgary, Alberta, Australia

## Abstract

Intestinal ultrasound (IUS) is emerging as a key tool to achieving the therapeutic target of transmural healing in inflammatory bowel disease (IBD). IUS is a non-invasive, radiation-free, imaging modality comparable to MRI, CT and ileocolonoscopy (IC). With the appropriate training and equipment, IUS can be an easily repeatable bedside test for IBD diagnosis and disease monitoring, including treatment response. Core to successful high quality IUS employment are appropriate training and expert techniques; however, the training pathway will not be explored in this review. Given the increasing shift towards objective assessment for tight disease control, gastroenterologist-led IUS should be incorporated into the armamentarium of imaging modalities alongside radiologists, to enhance our diagnostic and monitoring toolbox. This comprehensive review aims to outline the current literature around IUS and propose the placement of IUS in a treat-to-target algorithm in IBD. Ultimately, IUS facilitates timely management decisions to optimise patient care with potential to revolutionise patient outcomes, moving towards transmural healing as the holy grail of therapy in IBD.

## Introduction

Inflammatory bowel diseases (IBD), comprised of Crohn’s disease (CD) and ulcerative colitis (UC), are chronic immune-mediated gastrointestinal disorders characterised by periods of activity and remission. Diagnosis relies on clinical, biochemical, endoscopic and histological parameters.^1^ Intestinal ultrasound (IUS) exhibits increasing global uptake due to technological advancement, training opportunities and increasing expertise.^2–4^

The updated Selecting Therapeutic Targets in Inflammatory Bowel Disease (STRIDE II) guidelines raise treatment expectations. Therapeutic targets focus on clinical and endoscopic healing (EH), however, aspirational targets include histologic and transmural healing (TH) which reduce relapse rates, hospitalisation and need for surgery.^1^ Although ileocolonoscopy (IC) is the gold-standard for disease assessment,^5^ myriad limitations include need for anaesthesia/sedation, invasiveness, high costs with challenging access. Cross-sectional imaging has limitations: CT imparts radiation and requires iodine-based intravenous (i.v.) contrast, thus is not recommended for surveillance^5,6^ ; magnetic resonance enterography (MRE) involves long image acquisition times and often requires contrast.^6,7^ IUS presents a safe, accurate, cost-effective alternative for transmural assessment. Cost comparison of these modalities is however needed. IUS can be performed during gastroenterology assessment.^8–10^ This review aims to summarise how IUS is gaining recognition in IBD practice and illustrate IUS as an indispensable objective tool to guide therapy and limit disease progression and complications.^4^

## Imaging modalities in IBD: how does IUS compare?

Cross-sectional imaging is essential in IBD, as small bowel involvement is common, a ‘blind spot’ given limits in the reach of conventional endoscopy.^11^ Symptoms inconsistently predict endoscopic activity: up to 18% of symptomatic patients with CD lack ulceration at IC while some without symptoms display severe lesions.^12–14^ CT, MRI and IUS can be used to examine small bowel extent, skip lesions, strictures, fistulae or other penetrating complications.^5,15^ Emerging literature suggests all three have comparable diagnostic accuracy in diagnosis, therapeutic response and complication identification^16–21^ (Table 1). Notably, gastroenterologist-led IUS focuses on the intestine while other intra-abdominal/pelvic organs are not formally examined, a limitation that requires clear disclosure to patients. Where visualisation of the latter structures may be required, radiologic assessment should be sought formally.

**Table 1.** Advantages of IUS as point-of-care tool

Convenience Non-invasive Nil or minimal preparation Real-time results Low cost Ionising radiation-free In-patient and out-patient settings
Disease diagnosis Prompt identification of inflammation in work-up of gastrointestinal symptoms Accurate visualisation of intra- and extramural complications Refining diagnoses in work-up of gastrointestinal symptoms
Disease monitoring Early detection of inflammation during flare Discerning between flare and superimposed functional gut disorder
Disease severity Evaluation of fistulae, abscesses, and strictures Differentiation between abscesses and inflammatory masses
Disease therapy Assessment of therapeutic response to titrate ongoing medical management Facilitating early or prompt medical management

IUS, intestinal ultrasound.

### CTE

CTE has high accuracy for active small bowel inflammation, requiring oral and i.v. contrast.^22^ CTE detects mural healing and therapeutic response, with modest correlation with clinical, biochemical or endoscopic activity indices.^23^ Whilst CT detects small bowel pathology with equivalent sensitivity and specificity to MRI, radiation limits repeatability.^16,17,22^ CT/CTE is most relevant in acute or emergency settings, where urgent assessment of potential complications is warranted.^6^ Clinical and/or serological findings may guide appropriate CT/CTE utility to minimise recurrent radiation exposure.^5^

### MRE

MRE is preferred over CT without significant difference in disease localisation (*p* = 1.0), bowel wall thickening (BWT) (*p* = 1.0), wall enhancement (*p* = 1.0) or detection of fistulae (*p* = 0.08), lymphadenopathy (*p* = 1.0) and perivisceral fat enhancement (*p* = 0.31). Additionally, MRI is superior in per segment analysis in detecting ileal wall enhancement (*p* = 0.02) and strictures (*p* = 0.04).^22^ Ample literature supports high correlation between MRE and endoscopy in assessment of inflammatory IBD activity.^20,24,25^ MRE is however time-consuming and costly, somewhat limited by breath-holding ability and claustrophobia.^26^ Risks of contrast agents when employed, such as nephrogenic systemic fibrosis and substance accumulation within the basal ganglia have been documented, however, the former is usually minimised through routine renal function testing and appropriate patient selection, and the clinical significance of the latter remains unclear.^27^

### IUS

A landmark prospective multicentre trial comparing the diagnostic accuracy of MRE and IUS for the extent and activity of newly-diagnosed and relapsed CD (METRIC) confirmed both are accurate with high sensitivity for detecting small intestinal disease.^21^ MRE sensitivity was 97% (95% CI 91–99) for detecting terminal ileal disease with IUS sensitivity 91% (95% CI 79–97).^21^ Colonic disease is universally more challenging: MRE sensitivity was 41% [26-58] while IUS was 49% [33-65], not statistically significant.^21^ Unlike IUS, the accuracy of MR for distal colonic disease is enhanced with rectal contrast.^28^ Literature demonstrates comparable agreement between IUS and MRE in detecting IBD location and activity,^19,29^ although MRE is more sensitive in assessing disease extent.^19^

## Standard monitoring in IBD

Clinical, biochemical and endoscopic parameters are used to assess IBD activity and therapeutic response with a shift towards objective improvement to guide therapy.^1,5,30^ Notably, imaging cannot replace endoscopy and biopsies for dysplasia surveillance and tissue diagnosis.^5^

### Biochemical markers

C-reactive protein (CRP) >5 mg l^−1^ has high specificity for detecting endoscopic IBD activity but low sensitivity in excluding a flare.^31^ Faecal calprotectin (fcal), a neutrophil-derived protein with high sensitivity and low specificity for intestinal inflammation, correlates with endoscopic disease in IBD diagnosis, relapse and treatment response, particularly in the colon^5,32–34^ with values <150 μg/g suggesting endoscopic healing (EH).^1^ It is more specific and sensitive than CRP, unaffected by extraintestinal pathology.^35^ Abnormal CRP and fcal should prompt exclusion of infection and confirmation with endoscopy or imaging.^5^ Whilst blood and stool biomarkers are compositely used to predict disease activity and therapeutic response, they cannot predict disease location nor extent, and can be falsely negative.^1,36^

### Endoscopy

EH in CD is associated with improved long-term outcomes, reduction in bowel damage, relapse, surgery risk and complications even in clinical remission.^37,38^ IC or flexible sigmoidoscopy ± biopsies remain the reference standard for disease activity assessment.^5^ Mucosal healing (MH) is associated with long-term corticosteroid-free clinical remission, reduced risk of colectomy and inflammation at 5 years.^39,40^ Histologic healing is associated with reduced complications including hospitalisation, corticosteroid use^41^ and cancer prevention.^42^ However, histological targets are tempered by lack of established incremental gain over MH alone.^43^

Video capsule endoscopy (VCE) is an adjunctive modality for small bowel evaluation. VCE is well-tolerated, minimally invasive, without sedation requirement;^44,45^ however, access is not ubiquitous due to cost and local expertise.^46^ Patency capsule clearance may be necessary^5,45^ as VCE retention occurs in 2–13% due to stenoses/strictures.^47^ It is considered safe to proceed to VCE if the patency capsule is passed after 30 h or confirmed radiologically.^48^

## Advantages of IUS compared to other monitoring techniques

**Figure 1.**
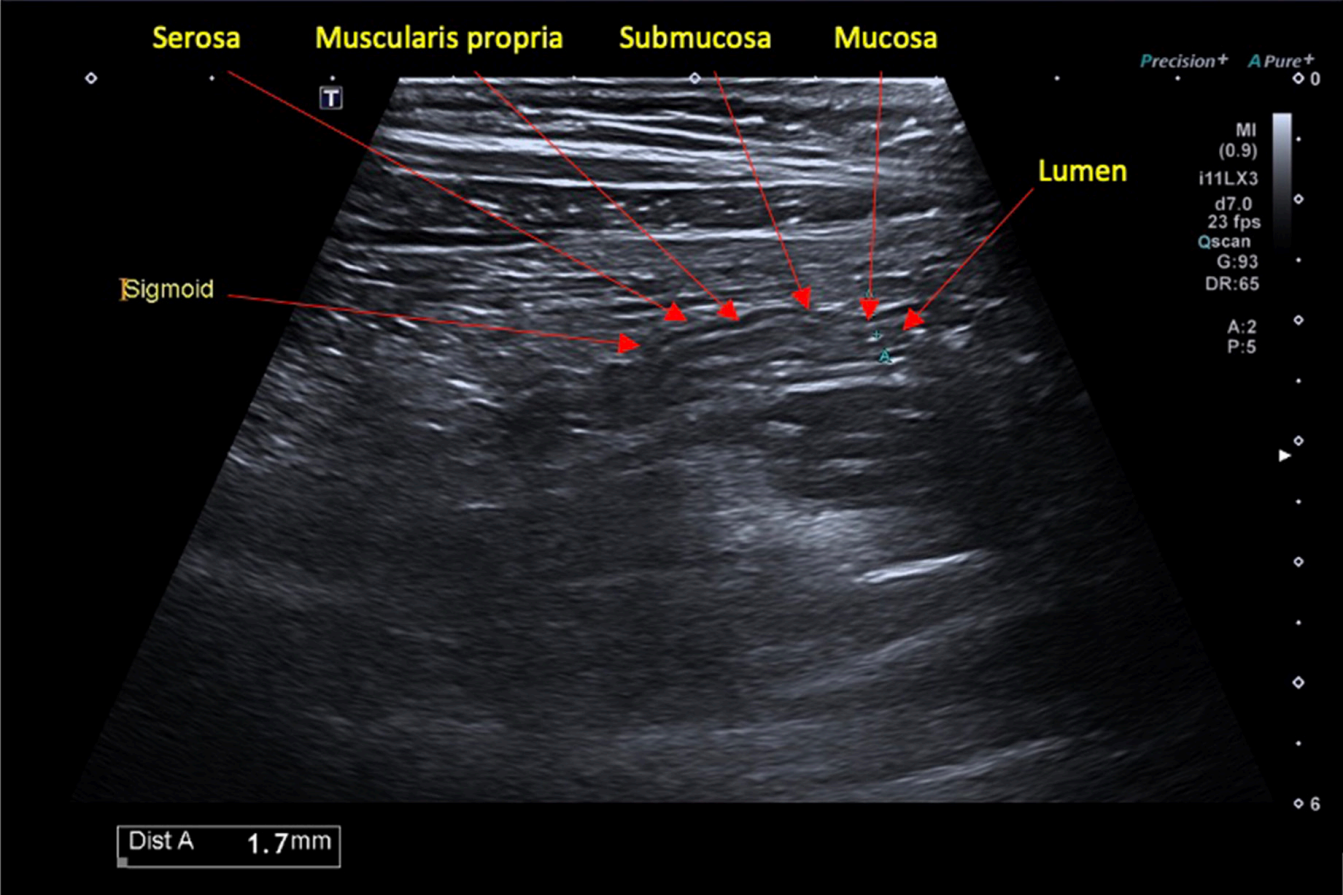
Normal BWT seen in longitudinal view of the sigmoid colon. BWT, bowel wall thickening

Transmural healing (TH) is an aspirational target for CD beyond endoscopic MH, imparting further beneficial outcomes^49^ with IUS well-placed to assess TH (Figure 1). Patient experience and preference are increasingly recognised in monitoring strategies, given the potential impact on adherence and outcome, yet have not directed algorithms nor guidelines to date.^5^ IUS is well-tolerated, easily repeatable with opportunity to engage patients in real-time.^50^ Otherwise, patients need to return to their provider for information.^35,51^ Bedside IUS facilitates timely medical decision-making^35,51^ a distinct advantages over MR/CT.^50^ Patients report enhanced disease understanding and confidence in making informed treatment decisions.^51^ Nearly all (99%) patients in the METRIC trial rated IUS as acceptable compared to 88% for MRE.^21^ IUS also exhibits high interobserver agreement;^51,52^ in the METRIC cohort, κ coefficient (k) was 0.64 for small bowel disease diagnosis and 0.63 for relapsing disease.^53^ In UC, interobserver agreement for BWT is almost perfect with intraclass correlation coefficient (ICC) of 0.96, with substantial agreement for colour Doppler imaging (CDI) *k* = 0.63, disease activity *k* = 0.77 and ICC of 0.93 for disease severity emphasising IUS reliability.^54^ Real-time interobserver agreement is also demonstrated in CD: six blinded operators exhibited moderate agreement on BWT and stratification, vascularisation, and lymphadenopathy and substantial agreement on lesion location, and presence of fistula, phlegmon and abscess. Not so for mesenteric adiposity changes, lesion extent, narrowing and prestenotic dilatation with poor agreement demonstrated.^55^ In certain populations, IUS may be less prone to technical difficulties compared with MRE, without motion artifact causing image degradation^29^ or bowel preparation.^50^ There are recognised challenges in performance of IUS, such as increased abdominal adiposity limiting resolution of bowel loops in addition to limitations in anatomic resolution.

## IUS findings

### Bowel wall thickness (BWT)

BWT is the most reliable and important measure of IBD activity with good interobserver agreement (k 0.72–1).^3,18,56^ BWT is measured perpendicular to the wall in axial and longitudinal axes, from the mucosal interface to the serosa, avoiding mucosal folds and haustra^3,4,57^ (Figure 1). A threshold value of >3 mm is agreed to be pathological, with a sensitivity of 88% and specificity of 93%, more accurate compared to a cut-off of 4 mm (75 and 97% respectively).^57,58^ While some advocate for a continuous scoring of BWT, others categorise, *e.g*. Ultrasound Global Assessment of Disease Activity Score, BWT of 4.0–6.0 is mild inflammation; 6.1–8.0 moderate; and >8.0 severe.^57^ BWT is considered a surrogate marker of transmural inflammation with thickening predictive of disease recurrence and surgery risk.^59,60^

### Bowel wall stratification (BWS)

**Figure 2.**
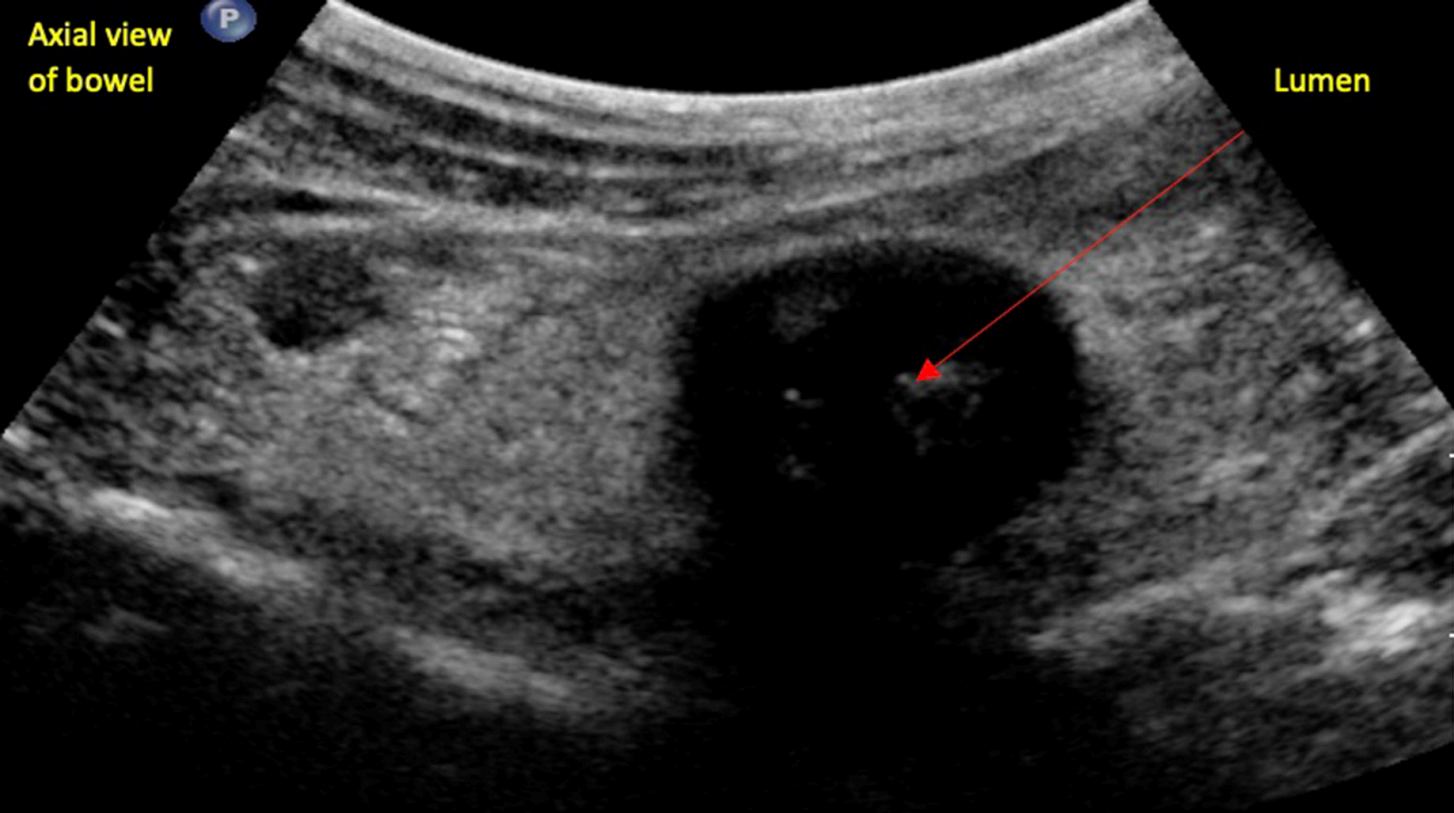
Complete loss of bowel wall stratification.

IUS is the only modality that clearly depicts wall layering^4,57^ (Figure 2). Interreader variability for detecting BWS is fairly reliable with k −0.22–0.85.^56^ Focal and/or extensive disruption in BWS correlates with active disease.^3^ Chronic inflammatory changes often lead to architectural alterations involving the intestinal submucosa, characterised by increased BWT and echogenicity.^57^

### Hyperaemia

**Figure 3.**
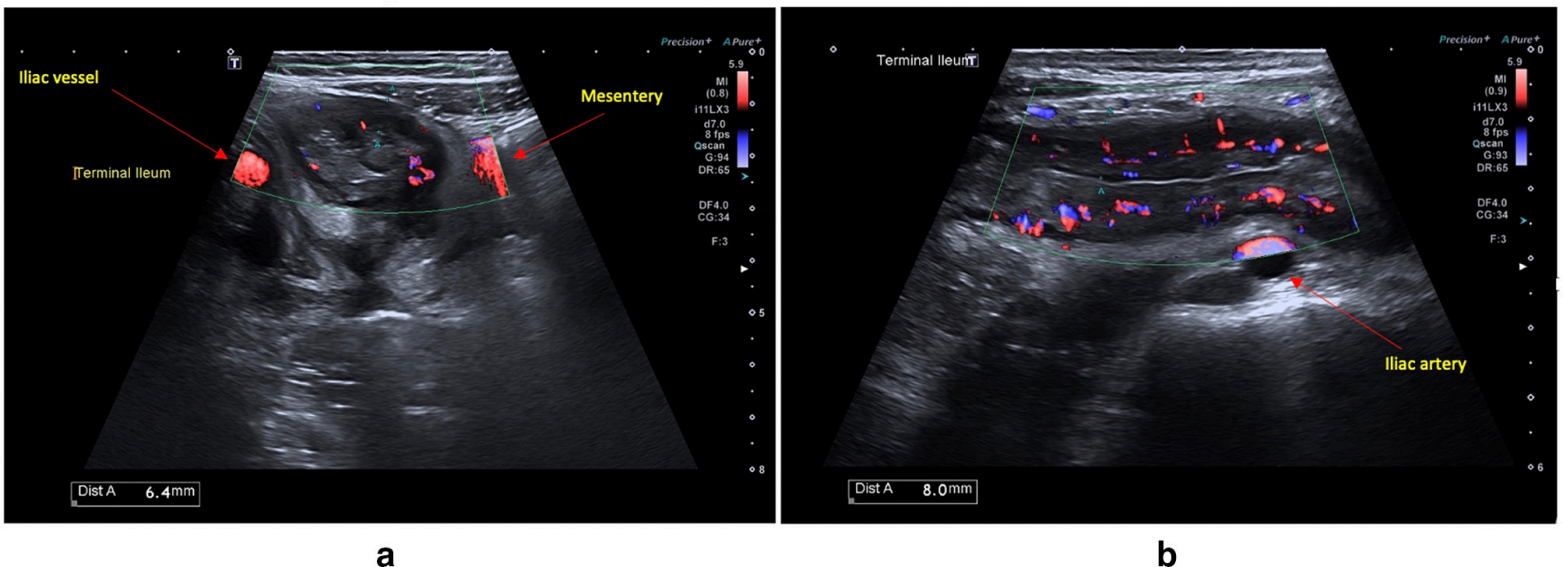
(**a**) Increased signal on colour Doppler imaging of the terminal ileum. (**b**) Grade 4 hyperaemia on Doppler with extension into mesentery.

Increased vascularity of the bowel wall due to inflammation is assessed by colour Doppler imaging (CDI), set to detect low flow, small calibre vessels.^57^ Normal bowel has minimal vascularity on CDI; however, increased intramural and extramural flow strongly correlates with histologic inflammation^61^ (Figure 3a and b) with grading as suggested (Table 2) . There is variable interobserver agreement with k 0.53–0.89,^56^ likely due to its semi-quantitative and subjective assessment, contingent upon machine settings and physiologic variability.^4^ Reliable depiction of CDI requires careful, consistent machine calibration, breath-holding, and standard machine settings.^63^ It is predominantly qualitative thus should be interpreted with some caution. Deep bowel loops may reveal falsely low signal, and inflamed bowel may not have detectable flow. However, CDI remains one of the most important parameters contributing to inflammatory activity.

**Table 2.** Limberg grade description^61,62^

Grade 0 No bowel wall thickening, no vascularisation
Grade 1 Bowel wall thickening, no vascularisation
Grade 2 Bowel wall thickening with short stretches of vascularity
Grade 3 Bowel wall thickening with long stretches of vascularity
Grade 4 Bowel wall thickening with long stretches of vascularity reaching into the mesentery

### Lymphadenopathy

Mesenteric lymph nodes (LN) are common yet non-specific, often present in healthy individuals, especially children.^4,64,65^ LNs > 10 mm (short axis) are more likely to be pathologic.^3,65^ Interobserver LN assessment is reproducible (k 0.56–0.90).^56^ However, the lack of association with clinical or biochemical activity and poor specificity excludes LNs from most activity indices.^63^

### Mesenteric fat hypertrophy (MFH)

Creeping fat or MFH due to fibro-fatty proliferation is important pathophysiologically,^66^ appearing as hyperechoic, homogeneous tissue on the mesenteric aspect of diseased bowel.^3,64,67^ Although it has poor interobserver agreement (k 0.14–0.69),^56^ MFH is associated most strongly with histological grade of inflammation compared with focal hyperechogenicity without fat wrapping or stratified pattern.^68^

### Extramural complications: strictures, fistulae, abscesses

**Figure 4.**
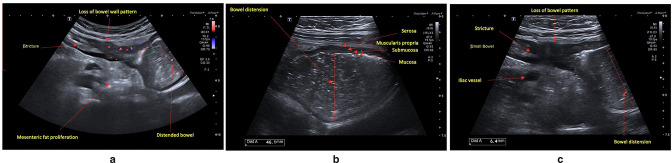
(**a**) Stricture with pre-stenotic features of hyperaemia, loss of wall stratification and marked mesenteric inflammatory fat (**b**). Dilated small bowel segment with stagnant fluid contents suggestive of bowel obstruction (**c**). Luminal narrowing with per-stenotic dilation and loss of wall stratification within a stricture.

Strictures are common in CD, leading to clinical and subclinical obstruction (Figure 4a, b and c). Defined as luminal narrowing, strictures exhibit increased BWT, pre-stenotic dilatation, with luminal diameter greater than 25–30 mm often associated with hyperperistalsis of the prestenotic segment.^3,69^ Early strictures may not exhibit proximal dilatation. Discerning the relative contribution of inflammatory *vs* fibrotic components (potentially reversible *vs* irreversible) remains challenging, impacting optimal medical *vs* surgical/endoscopic dilation intervention.^70^ IUS techniques are rapidly evolving to address this conundrum: CDI, elastography, echostratification and contrast-enhanced ultrasound (CEUS) may provide valuable insight.^3,71^ Strictures associated with inflammation may exhibit BWS loss and hyperaemia. Stratification loss may also signify smooth muscle hypertrophy, refractory to medical therapy (CDI may help to discern the two); fibrotic strictures can exhibit echogenicity and hypovascularity portending need for endoscopic dilatation or surgery.^3,64,72,73^ IUS detection is highly accurate compared to CT/MRI and gross pathology: pooled sensitivity of 79% (95% CI 71–84%) and specificity 92% (95% CI 87–96%).^9,74^ IUS also affords real-time assessment of intestinal motility, distinguishing collapsed bowel or functional contractions in strictures and dysmotility associated with obstruction.^64^

**Figure 5.**
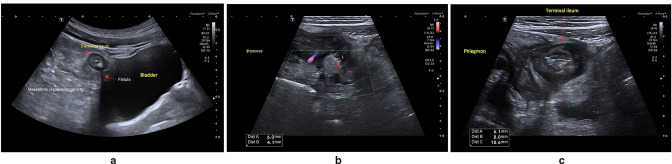
(**a**) Enterovesical fistula with echogenic inflammatory fat. (**b**) Penetrating terminal ileal disease with hypoechoic area (6*4.1 mm) suspicious for abscess with marked inflammatory for echo. (**c**) Severs increase in BWT in the terminal ileum hypoechoic area deep to inflamed loop suggestive of phlegmon. BWT, bowel wall thickening.

IUS can detect fistulae, sinus tracts and fissures^3,57,64^ with similar accuracy to CT and MRI^22,75^ (Figure 5a). Fistulae result from extramural fissures arising from deep intestinal ulcerations communicating with other tissues, whereas sinus tracts are linear extensions/blind-ends.^3,64^ Both sinus tracts and fistulae are hypoechoic irregularities arising from thickened bowel with or without gas, with a diameter <2 cm.^3,19,76^ In one systematic review, IUS had a pooled sensitivity of 74% (95% CI 67–79%) in detection of enteric fistulae compared with surgery.^16^

Compared with surgery, IUS has a pooled sensitivity of 84% (95% CI 79–88%) and specificity 93% (95% CI 89–95%) for abscess detection.^16^ Microperforations may appear as inflammatory perienteric masses without extraluminal air (phlegmon). Spiculation or hypoechoic stranding from the outer serosa^57,77^ are harbingers of penetrating complications: abscesses (Figure 5b) or poorly defined inflammatory masses (Figure 5c): hypoechoic masses with or without echogenic gas.^57^ An inflammatory mass can be differentiated from abscess with CDI or CEUS.^3,57,64^ Interobserver agreement is excellent for detection of stenosis in IBD using IUS (k 0.81–1), whereas for fistulae it varies.^56^

IUS can characterise small bowel motility in real-time although standardisation is needed. Peristalsis is non-specific, however, can be reduced or absent in diseased bowel. Free intra-abdominal fluid juxtaposed to diseased bowel is non-specific but can reflect disease severity.^3,64^

## Utility of IUS in CD

### Diagnosis, disease extent and activity

Although important for quality of life, clinical symptoms correlate poorly with objective inflammation. In the CALM trial, use of symptoms to guide treatment in CD resulted in lower rates of EH compared to patients with composite clinical and biomarker assessments.^36^ IUS is comparable in diagnosing CD compared to MRE and IC^19,21^ with diagnostic sensitivity of 94%, specificity of 97%, a positive-predictive value of 97% and negative-predictive value of 94%.^19^ The highest diagnostic performance is in the ileum, sigmoid and descending colon, with lower accuracy in the duodenum, proximal jejunum and rectum.^10,78^ Allocca et al^52^ showed IUS had 92% sensitivity and 100% specificity for presence of ulcers at colonoscopy, with high diagnostic accuracy compared to MRE and IC: 91% for disease localisation, 96% for ulceration.^52^ Repeatability during follow up and timely therapeutic response measurement is key: Kucharzik et al.^67^ conducted the multicentre TRUST study, serially following CD patients over 12 months demonstrating IUS changes in response to therapy.^79^

### Post-operative recurrence

IUS confers high accuracy in detecting recurrence compared to IC,^64,80^ whereas clinical and biomarkers correlate poorly with IC.^81^ Although fcal is accurate, a moderate false-positive rate in discerning post-operative recurrence exits^82^ which may improve when combined with IUS.^64^ BWT correlates with endoscopic recurrence and lack of therapeutic response signifying surgical risk.^59,83,84^ BWT > 3 mm had 79% sensitivity and 95% specificity for post-surgical endoscopic recurrence (Rutgeerts’ score ≥i2): sensitivity 93% for detecting severe post-surgical recurrence (Rutgeerts’ score ≥i3).^60^

## Utility of IUS in UC

### Diagnosis, disease extent and activity

Evidence for IUS in UC is evolving with IC and biopsies the mainstay of UC diagnosis.^3,5,20,85^ In UC, symptom correlation is still imperfect.^1,86^ Persistent, low-grade inflammation portends worse outcomes, including hospitalisation and corticosteroid need.^41^ IUS can be used to detect BWT in active UC^3^ although absence of BWT does not exclude active inflammation, and CDI is an important tool to detect mild to moderate inflammation limited to the mucosa. It is accurate in detecting inflammation and defining extent proximal to the rectum.^3,87^ TRUST&UC, a multicentre prospective observational study demonstrated IUS as a sensitive tool detecting disease activity with increased BWT in 88.5% of those flaring with left-sided or pancolitis.^35^ Whilst rectal assessment can be challenging, a transperineal approach is promising^88^ and IUS remains valuable conferring more than 70% sensitivity in detecting disease proximal to the rectum, with up to 97% sensitivity in the sigmoid and descending colon.^10^ Loss of BWS, increased hyperaemia and MFH are also features of greater disease activity.^4,10,35^

The TRUST group demonstrated BWT and CDI mirrored improvements in clinical scores and fcal at 3 months, with changes seen as early as 2 weeks.^35^ This rapid IUS response to intensified therapy with subsequent response on clinical indices cements the role of IUS in UC monitoring.^35^ Early evidence also predicts corticosteroid response in guiding salvage therapy timing in acute severe UC.^89^

## Adjunctive techniques in IUS

### Small intestine contrast ultrasonography (SICUS)

Ingestion of a neutral oral contrast agent, (500–800 ml polyethylene-glcyol/PEG) 30 min pre-examination increases sensitivity for detection of proximal small bowel disease, strictures and penetrating complications.^3,64,76^ Pallota et al showed SICUS had 97.5% sensitivity and 100% specificity in identifying at least one stricture (*k* = 0.93) and sensitivity of 75 and 100% specificity for at least two or more strictures (*k* = 0.78).^76^ Additionally, SICUS had 96% sensitivity and 90.5% specificity (*k* = 0.88) in identifying fistulae in 27/28 patients as well as 100% sensitivity and 95% specificity (*k* = 0.89) in detecting intraabdominal abscesses in 10 patients undergoing bowel surgery.^76^ Despite this, oral contrast is not commonly employed clinically, as it prolongs exam time and is not preferred by patients.

### Transperineal ultrasound (TPUS)

A small high frequency curvilinear or linear transducer on the perineum can visualise the anal canal and surrounding soft tissues to characterise penetrating, perianal disease.^57,64^ Presence of internal and external fistulae, location within the anal canal and clock-face representation can be accurately documented.^57^ TPUS has high diagnostic accuracy compared with both endoanal ultrasound: sensitivity of 84.9% for fistulae detection^90^ ; and MRI, with a sensitivity of 90.6%, with excellent agreement between TPUS and MRI (*k* = 0.783).^91^ The accuracy in detecting abscesses has pooled sensitivity of 86% (95% CI 67–99%) and positive-predictive value of 90% (95% CI 76–99) in a meta-analysis.^92^ Whilst TPUS is currently useful in screening for perianal disease, its role in assessing disease severity and monitoring response remains to be clarified, and operator expertise must be acknowledged.^92^

### Contrast-enhanced ultrasound (CEUS)

Although CEUS utility with standard greyscale B mode IUS for luminal assessment of disease activity is limited globally, it can aid in differentiation between an inflammatory mass *vs* abscess. IUS with microbubble contrast, comprised of 2–6 μm lipid-coated gas particles^57^ is used to augment perfusion assessment. A retrospective review demonstrated 100% specificity for abscess detection among 71 inflammatory masses in 50 patients, resulting in *k* = 0.972 for differentiating abscess *vs* phlegmon.^93^ The role of CEUS in activity assessment is contentious, with lack of agreement on peak enhancement or area under the curve thresholds, adding significant complexity to standard examination, limiting uptake.

### Shear wave elastography (SWE)

SWE is an exciting IUS application measuring shear wave speed generated by an acoustic pressure wave through bowel as a marker of tissue density and stiffness in real-time,^57,64,94^ reflecting fibrosis *vs* inflammation in strictures. SWE is higher in fibrosis with AUC of 0.822 when using cut-off 22.55 kPa as a discriminator between mild-moderate and severe fibrosis.^95^ Combined with CEUS, SWE can accurately identify inflammation and smooth-muscle hypertrophy.^95^ However, its application is limited as standardisation of measures across machines is lacking.

## IUS activity scores: utility and limitations

There is no consensus on an optimal IUS scoring index.^96^ The recently derived International Bowel Ultrasound Segmental Activity Score (IBUS-SAS) uses reliable sonographic components including BWT, BWS, CDS and inflammatory fat.^63^ The Simple Sonographic Score comprises BWT and Doppler,^97^ developed using statistically significant parameters in a retrospective then prospective evaluation of IUS with endoscopy with an AUROC of 0.836 when applied to a prospective cohort for external validation.^97^ Due to lack of prospective validation, widespread use of scores is limited. Currently, a large international multicentre trial is in progress to establish this.^98^

## Targeting transmural remission (TR): IUS in clinical practice

**Figure 6.**
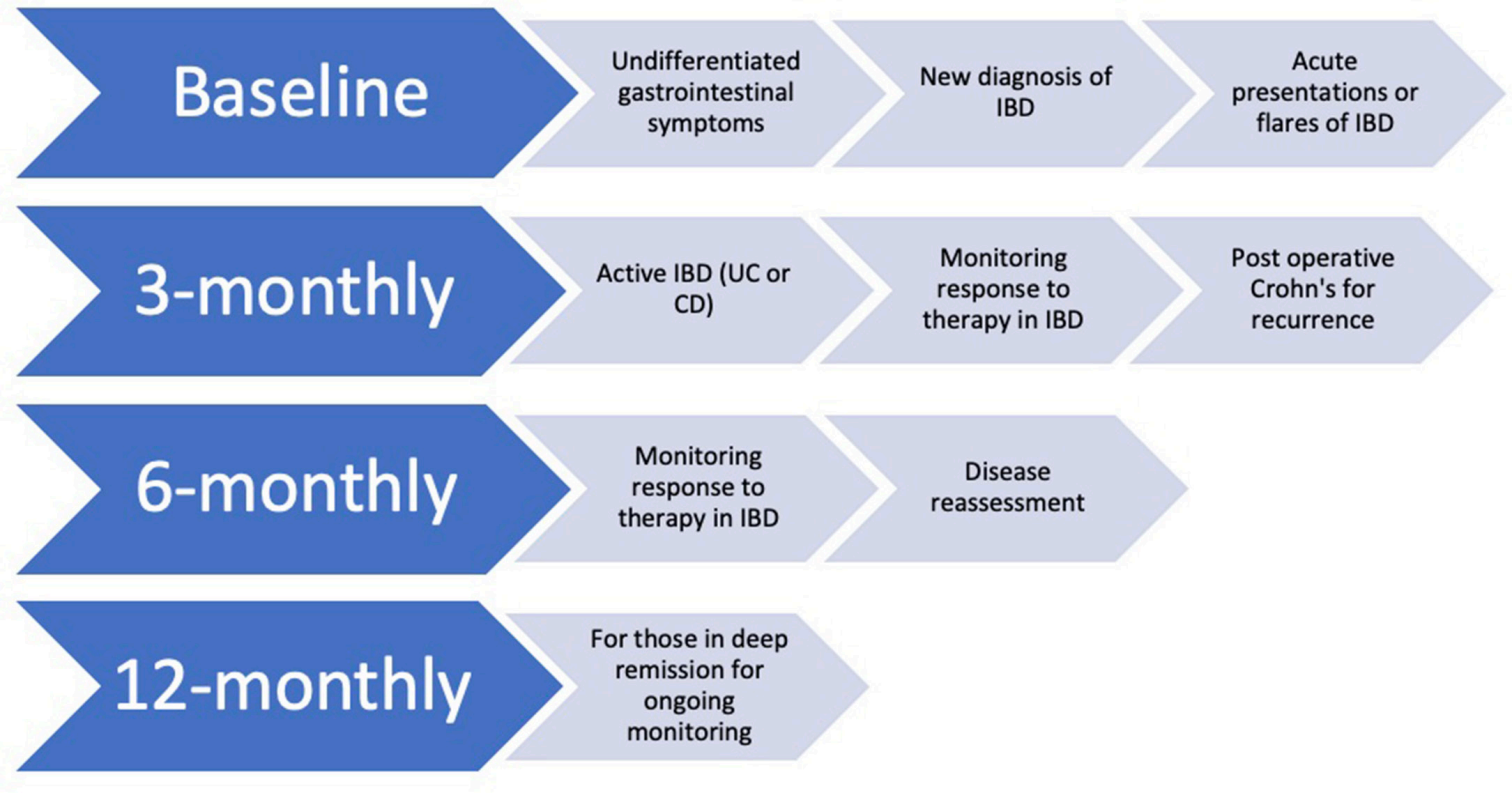
Timing of IUS. CD, Crohn’s disease; IBD, inflammatory bowel disease; IUS, intestinal ultrasound; UC, ulcerative colitis.

Transmural remission (TR), defined as the resolution of mucosal ulcerations, transmural disease and extramural disease^99^ is associated with sustained clinical remission, reduced need for medical escalation, surgery and prevention of bowel disease progression with better outcomes than endoscopic remission^49,100,101^ (Figure 1). We propose an algorithm for timing IUS at index, followed by routine surveillance^36^ (Figure 6). IUS performed at baseline for those with suspected IBD or symptoms, as recommended by ECCO-ESGAR, to ensure exclusion of complications and proximal disease in IBD.^5^ In established IBD, serial exam intervals are contingent upon treatment trajectory: to evaluate therapeutic response, after drug initiation, evaluation at 12 weeks is ideal. Earlier evaluation may be important as the TRUST UC showed changes at 2 weeks.^35^ Post-operative IUS should occur within 3–6 months with fcal, which may obviate endoscopic confirmation and optimise resource use. Use of MRE/CTE is important where IUS is limited or for complex perianal disease, complex post-operative anatomy or need for exclusion of extraintestinal manifestations, such as sacroiliitis. IUS should be correlated with biomarkers to objectively guide timely clinical decisions. Presence of complications may necessitate additional imaging such as MRE prior to endoscopic or surgical intervention.

### What is the treatment target in IUS and is it achievable?

**Figure 7.**
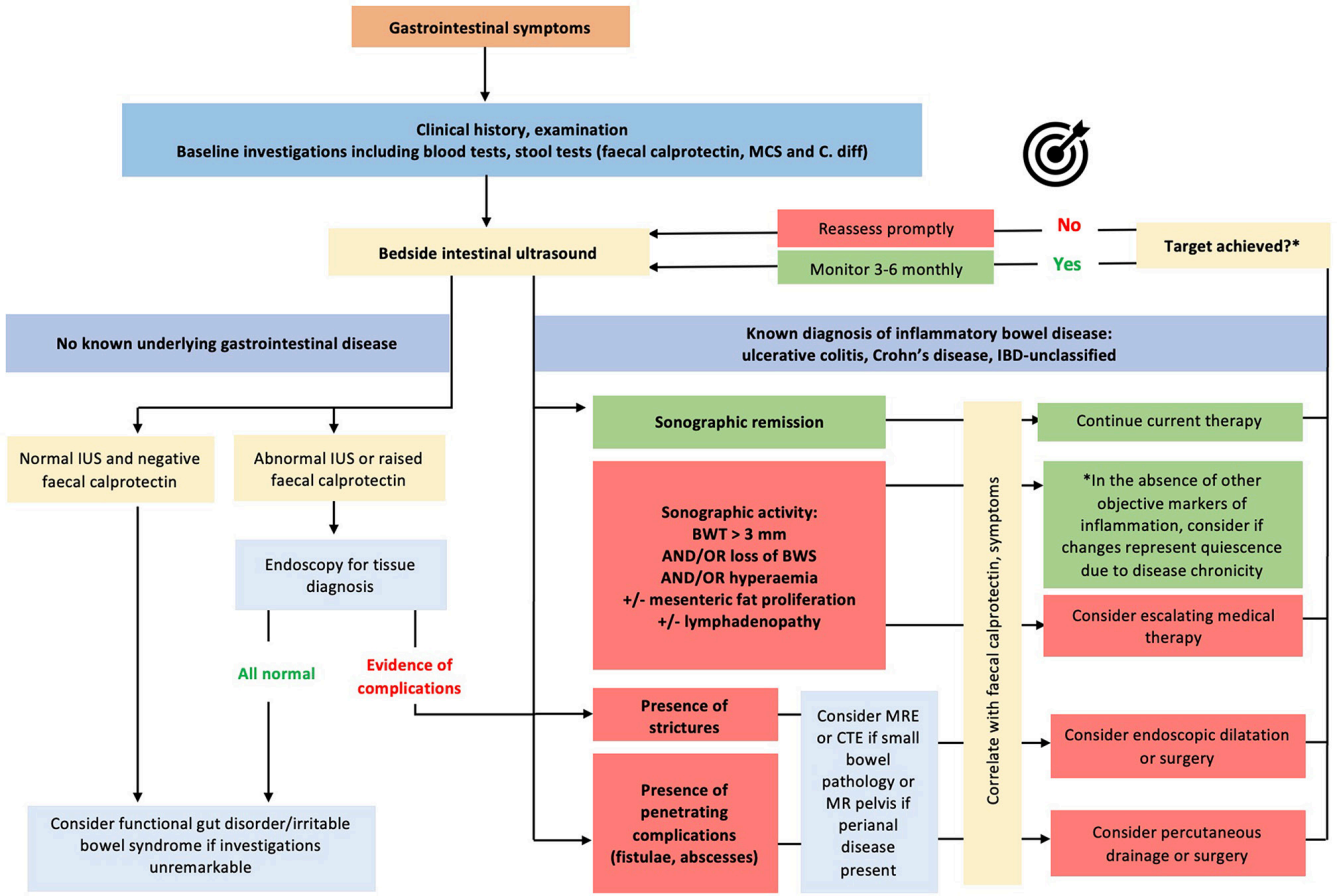
A proposed algorithm on how to incorporate IUS in treat to transmural healing. BWT, bowel wall thickening; CTE, CT enterography; IBD, inflammatory bowel disease; IUS, intestinal ultrasound; MRE, magnetic resonance enterography

A recent systematic review established much needed expert consensus on IUS treatment response and transmural remission.^102^ The presence of complications including stenoses portends poorer response and clinical outcomes compared to uncomplicated luminal inflammatory disease, likely related to the reversibility of disease and respective damage.^102^ High quality, large population studies are needed regarding monitoring and resolution of CD-related intestinal complications. Colonic disease tends to respond faster than ileal disease.^102^ Another important reportable aspect of response is reduction in length of disease, in addition to improvement in BWT (>25%), BWS, hyperaemia, mesenteric inflammatory fat proliferation, and lymphadenopathy.^35^ Incremental levels of healing offer superior outcomes in IBD, although a minority of patients will achieve deeper TH (~25%) and histological healing (~10%) with conventional therapy (Figure 7).

## Future directions

Despite its paradigm-changing potential, widespread uptake of IUS remains challenging.^64,103^ Incorporation of IUS into routine IBD practice requires specialised training, service delivery adaptation, with investment into ultrasound technology and funding model evolution.^103^ Current funding models favour MRE or CTE. Coordinated efforts between national and international groups beyond Europe are needed to encourage IUS in guidelines for IBD monitoring and must include patient experience and preference in their consensus.^64,103^ Broadening IUS applicability to other intestinal disorders has been suggested given its timely nature including functional motility gastrointestinal disorders and visualisation of faecal loading,^64^ however further prospective studies are warranted to validate such use.

## Conclusion

IUS is integral to future patient-centred, innovative models of IBD care. Monitoring strategies should align with patient preferences and foster patient engagement. Multidisciplinary care is the foundation of excellence in care, combining expertise in imaging from both specialist gastroenterologists coordinating medical care with experts in diagnostic imaging, to leverage the benefits of all dedicated small bowel imaging modalities. Integration of IUS into clinical practice will facilitate early and accurate IBD disease assessment to enable objective monitoring and expedited therapeutic decision-making, improving patient experience, outcomes and quality of life.
